# Student interest, concerns, and information-seeking behaviors related to COVID-19

**DOI:** 10.1186/s43031-022-00053-2

**Published:** 2022-04-04

**Authors:** Jamie N. Elsner, Troy D. Sadler, Laura Zangori, Patricia J. Friedrichsen, Li Ke

**Affiliations:** 1 School of Education, University of North Carolina at Chapel Hill, 100 E Cameron Ave, Chapel Hill, NC 27514 USA; 2 College of Education, University of Missouri, 611 Conley Ave, Columbia, MO 65211 USA; 3 College of Education and Human Development, University of Nevada, 1664 N. Virginia St, Reno, NV 89557 USA

**Keywords:** Student interest, Information sources, Science learning, COVID-19

## Abstract

COVID-19 creates an opportunity for science classrooms to relate content about viruses to students’ personal experiences with the pandemic. Previous researchers have shown that students are interested in crisis situations like disease outbreaks; however, they primarily acquire information about these events through internet sources which are often biased. We argue that it is important to understand student interest, concerns, and information-seeking behaviors related to COVID-19 to support science classroom learning and engagement about the virus and other potential outbreaks. We surveyed 224 high school students and analyzed their responses to six open-ended questions. We found that students expressed the most interest in topics related to the origin of COVID-19 and vaccines. Their greatest concerns included contracting the virus or someone they know contracting the virus and vaccine distribution. Of our sample, only 6.7% reported using their teachers as their source of COVID-19 information. Science classrooms have the potential to pique students’ situational interest by discussing COVID-19 topics that are important to students, which can increase their academic performance, content knowledge, attention, and engagement in learning about viruses. Moreover, classroom instruction about COVID-19 by teachers has shown to alleviate students’ stress and anxiety. We provide key areas of student interest about COVID-19 to help educators address students’ questions and improve curricular resources on viral pandemics.

## Introduction

The COVID-19 virus has spread rapidly across the world, totaling over 364 million confirmed cases and 5.6 million deaths globally (WHO, 2022). Crisis situations, such as this disease outbreak or natural disasters, profoundly impact students’ lives; moreover, researchers have shown that student understanding of these events is based largely on personal experiences rather than formal knowledge (Lee, 1999). For instance, Cvetković et al. (2015) found that secondary students in Serbia who experienced earthquakes were more likely to know what constitutes an earthquake compared to students who had no personal experience with earthquakes. Furthermore, students with an understanding of earthquakes expressed more interest in expanding their knowledge about earthquakes (Cvetković et al., 2015). Similarly, this pandemic presents an opportunity for students to relate their lived experiences during COVID-19 to science classroom instruction about the virus. We contend that it is essential to understand student interest about COVID-19 to support learning as students develop more complex scientific knowledge about the pandemic.

While much of current research in education during the pandemic has focused on students’ attitudes, mental health concerns, and the impact of school closures (Mirahmadizadeh et al., 2020; Rao & Rao, 2021; Thakur, 2020), little is understood about high school students’ interest in learning about COVID-19. The benefits of student interest on academic achievement and learning have been well-documented (Hidi & Renninger, 2006; Schiefele et al., 1992). In a recent study, Gustia and Suhartini (2021) found that higher levels of interest in learning about biology during COVID-19 significantly increased students’ cognitive outcomes for biology. Yet, the specific topics of interest to students related to COVID-19 have not been explicitly explored. There remains a need to document the questions, concerns, and topics of interest that students have in order to help educators foster students’ scientific curiosities and further develop their understanding of the pandemic (and future pandemics).

In addition, knowing where students acquire information about the topics that interest them can provide insight into the accessibility and comprehensibility of different forms of media for students. For example, some students may seek video sources like Youtube or TikTok rather than text-based information. In the digital era, students are exposed to a vast array of COVID-19 related information sources—tv news, social media, online news—with varying degrees of credibility. This makes deciphering factual versus fictitious information challenging for students. In a survey of Indonesian Generation Z students born from 1995 to 2010, social media and news websites were the most common sources of health information during the COVID-19 pandemic (Roselina et al., 2021). Importantly, 45% of respondents expressed uncertainty over the validity of the information they acquired. Classroom discussion around the most used information sources can help students identify biases in the media they consume and expand their digital literacy skill set. In turn, students will be better equipped to make informed decisions regarding their health such as wearing masks, social distancing, and getting vaccinated.

Connecting students’ interests about COVID-19 to classroom instruction allows students to apply their funds of knowledge to science content about the virus. Funds of knowledge validates the personal experiences, beliefs, values, culture, and traditions that students hold as knowledge resources in the classroom (Gonzalez et al., 2005). For this reason, we sought to understand students’ concerns about the pandemic and the impact of COVID-19 on their daily lives to contextualize student interest within their broader community and lived experiences.

Classroom discussion related to the COVID-19 pandemic is relevant to students’ lives, presents complex issues that are influenced by social factors like politics and economics, and requires negotiation of science as it connects to authentic problems in society. These criteria make COVID-19 instruction an ideal opportunity for science teachers to implement a socio-scientific issues (SSI)-based teaching approach (Zeidler, 2014). SSI-based teaching positions students to reason about and apply scientific principles to open-ended problems that lack obvious solutions and cannot be solved by science alone. By engaging with SSI, students develop an understanding of science content that have implications for society. Additionally, there is a positive association between SSI-based instruction and generation of student interest (Sadler & Dawson, 2012). However, many teachers do not feel well-supported in the selection and design of SSI-based curriculum (Tidemand & Nielsen, 2017). We provide key topics of interest related to COVID-19 that educators can leverage in their development and implementation of material on viral pandemics.

This study was guided by the goal of exploring students’ interests, ideas, and media choices relative to a pandemic through which they were living. We pursued this goal by answering the following four research questions.

1. What interests do high school students have about COVID-19 and how do interests change over the course of the pandemic?
2. Where do high school students acquire information about COVID-19 and how do their preferences for information sources change over the course of the pandemic?
3. What are high school students’ concerns about COVID-19 and how do their concerns change over the course of the pandemic?
4. How has COVID-19 impacted high school students’ lives and how do these impacts change over the course of the pandemic?

## Theoretical Framework

Our work is informed by Silvia’s (2006) framing of interest as an emotion that motivates individuals to cultivate knowledge about a particular topic. Educational researchers distinguish between two main types of interest when considering student engagement in learning: situational interest and individual interest. Situational interest refers to an individual’s focused attention and reaction triggered by external stimuli, which are often temporary (Nieswandt, 2007). In contrast, individual interest is an enduring, intrinsic predisposition for activities and tasks and has been shown to be strongly linked to academic performance (Hidi & Renninger, 2006; Schiefele et al., 1992). Individual interest is also associated with greater attention, goal setting, and engagement in learning (Hidi & Renninger, 2006).

Hidi and Renninger (2006) suggest that situational interest is a precursor to well-developed individual interest. Further, they recommend identifying areas of student interest early on to support the development of more complex individual interest. In the context of COVID-19, students experience the pandemic in almost every aspect of their daily lives and are bound to relate personal experiences to their classroom instruction. It is also important to acknowledge that engaging with media around the pandemic is part of students’ personal experiences. With the proliferation of personal computing devices (e.g., cell phones) and media platforms (including social media and more traditional outlets), there is a greater media influence within the lives of youth than ever before (Twenge et al., 2019). For this reason, we chose to explore questions related to students’ interests as well as their media preferences in the context of COVID-19.

## Literature Review

Researchers have shown that students demonstrate curiosity in their classrooms about crisis situations. Smith et al. (2017) surveyed approximately 2500 science teachers about whether and how they addressed the 2014 Ebola crisis in their instruction. Seventy-nine percent of the participating teachers reported that their students came to class with questions about Ebola, suggesting strong interest among students about the crisis. The interest demonstrated by students impacted classroom decisions; 95% of the teachers who reported teaching about Ebola indicated that their students’ interest in the outbreak was a motivating factor for teaching about the topic. Our research complements this study by focusing on what interests students demonstrate, as opposed to teachers’ perceptions of student interests, about viral pandemics to help educators and curriculum developers create more student-centered materials about COVID-19 and other pandemics.

In addition to interest, it is essential to know what sources of information students use to acquire information about COVID-19 due to the increasing frequency of false claims about the virus. The spread of misleading health information online is not novel to COVID-19. Social media was central to propagating misinformation during prior Zika, Ebola, and yellow fever disease outbreaks (Krittanawong et al., 2020). In the case of yellow fever, researchers found that 61.3% of tweets contained misleading information, such as medically inaccurate homeopathic treatments. Misleading tweets and conspiracy theories posted by grassroots users were more likely to be retweeted (Ortiz-Martínez & Jiménez-Arcia, 2017; Vijaykumar et al., 2018). Furthermore, Tran and Lee (2016) documented that the spread of Ebola-related information is largely determined by social ties—that is, a user was more likely to post about Ebola if they followed someone who had previously posted about it.

Although students share information via their social networks, they are not relying on their academic connections such as teachers as sources of information. A recent paper by Ho et al. (2020) surveyed approximately 2000 participants, 80% of whom reported internet media as their primary source for COVID-19-related information while only 20% relied on academic courses. However, those who received information from academic sources reported less worry than those who received information through internet media, traditional media, or friends. This research suggests that teachers could help alleviate students’ stress and worry about COVID-19 by discussing disease outbreaks in class.

Moreover, it is important to note that as interest increases about a topic so do the number, specificity, and complexity of sources that individuals rely on for information (Reagan, 1996). If teachers specifically address COVID-19 topics of high interest to students, then students will be more likely to distinguish the credibility of sources as their information repertoire grows. We know that young people have a desire to engage in digital citizenship that helps “contribute to, and inform, public dialogue” through their social media accounts (Gleason & von Gillern, 2018, p.210). For example, secondary students shared opinions and information on Twitter to raise awareness about gay rights, feminism, and political issues (Gleason & von Gillern, 2018). Teachers are positioned to connect students’ out-of-school civic engagement on social media to formal citizenship curricula related to the pandemic. In the context of COVID-19, science educators could integrate students’ social media literacy in discussion on collective responsibility and the science behind community health practices like social distancing and wearing masks. Because individuals passively absorb media that does not directly relate to one’s situation (Grunig, 1979), it is important to identify information sources that could pique students’ situational interest, thus leading to their active engagement in learning about the pandemic.

## Methods

When the COVID-19 pandemic first emerged in spring 2020, we began working with a group of high school teachers to explore ways in which COVID-19 could be productively incorporated into science classes (Sadler et al., 2020). Ultimately, we collaboratively developed an SSI unit that used COVID-19 and the question of how to slow the spread of the virus within students’ communities as the focal issue (Sadler et al., 2021). We worked with six of the teachers involved in these efforts to collect the data presented in this study.

### Data Collection

We designed an online survey consisting of multiple sections aligned with the research questions. Survey development began with a review of previously validated instruments for measuring student interest in science and technology (Romine et al., 2014; Romine et al., 2017). Some items were modified from these existing instruments, but we determined that most of the survey items (including all of the items used for data collection in the current study) would need to be newly created to ensure alignment with the research goals which related specifically to students’ interests and experiences with COVID-19. Three members of our research team (one researcher who has developed several instruments, another who specialized in qualitative research, and a third who had recent high school science teaching experience) created items for inclusion in the new instrument. The items were reviewed by other members of our team (including individuals with research and teaching expertise) and an external evaluator who has expertise in development and validation of research instrumentation. Following two iterations of review and revision with our research team and evaluator, we shared the instrument with three high school students and nine high school science teachers, who provided feedback and suggestions for slight revisions. Because we wanted to collect data as quickly as possible in order to capture student responses at the beginning of the pandemic, we did not take the additional step to pilot test the instrument.

Six of our partner teachers administered the survey *prior to* enactment of the COVID-focused SSI unit. The surveys were distributed at three time points in spring 2020, fall 2020, and spring 2021. Unique student samples completed the survey at each time point. In this paper, we focus on the students’ responses to five open-ended survey items:

1. What interests you most about the coronavirus?
2. What pieces of information about the coronavirus are you most interested in learning?
3. What sources of information are most important for you if you are trying to find out about an issue like the coronavirus?
4. What are your top three concerns about COVID-19?
5. How is COVID-19 affecting you and your family?

### Student Participants

Participants attended one of four high schools in a Midwestern state in the U.S. The schools were situated within urban and suburban communities. Among the surveyed students, 224 provided responses to the open-ended questions which included 74 students in spring 2020, 92 students in fall 2020, and 58 students in spring 2021. Of the full sample, 59.4% were women, 37.1% were men, 0.8% identified as transgender, 0.4% identified as non-binary and 2.2% did not identify with the gender categories provided. When asked about their race and ethnicity, 66.1% of students indicated White/Caucasian, 29.5% African American, 7.4% Latinx, 6.3% Asian, 1.3% Native American or American Indian, and 4.0% did not identify with the racial categories provided. Several students identified with multiple race options. Of the respondents, 7.6% were high school freshmen, 58.5% sophomores, 20.1% juniors, and 13.8% seniors.

### Analysis

We used a thematic analysis approach guided by the constant comparative method to analyze the data (Hatch, 2002). First, we open-coded students’ responses within each of the four major topics outlined in the research questions (i.e., interests in COVID-19, preferred information sources, concerns about COVID-19, and impacts on their lives). Two researchers worked together in the open coding phase through three rounds on analysis in which emergent codes were identified and student responses were sorted into these codes. After these initial rounds of open coding of the full data set, we examined all of the emergent codes to look for potential overlap and ways to condense the codes into a taxonomy of findings. This process of code combination and pruning resulted in the final set of codes which are presented in the results section. Once the final codes were established, the two coders assigned each student response to these codes, and there was 100% agreement of assignment of individual responses to particular themes. As a part of this process, we established trustworthiness through triangulation of multiple raters and peer debriefing (Lincoln & Guba, 1985).

Next, we quantified the frequency of the themes. Since items 1 and 2 both related to student interest and elicited similar student responses, we combined those items for the analysis. For example, if a student expressed the same theme in her response to item 1 and item 2, we only counted the theme once. To determine the overall percentage of students who expressed each theme, we added the frequencies across each time point and divided by the total number of respondents (*N* = 224). Only themes with greater than 11 total responses (5% of the sample) were included. Exemplar student responses were selected to demonstrate each theme.

Lastly, we ran chi-square tests for each of our identified themes to determine whether they changed across time points. If cell frequencies were less than five, we ran Fisher’s Exact Tests instead of the chi-square so as not to violate assumptions of the chi-square test. Temporal analysis across the three time points provided insight into the flow of student interest as opposed to total frequency.

## Results

This study examined student interest and information seeking preferences for COVID-19 at the beginning of and during the global pandemic. In addition, we documented student concerns about the crisis and its overall impact on students’ daily lives. The results are presented in order of the research questions. Our presentation of each research question includes a data table, which displays the themes we identified, a description of each theme, an exemplar student response that demonstrates the theme, and the overall percentage of respondents who expressed the theme in their responses. While theme percentages were low overall, this was expected as the survey items were open-ended, and students raised points on their own without being prompted. Therefore, we view themes with greater than 11 responses or 5% of the sample as high.

### Student Interest

We identified nine COVID-19 interest areas among high school students (See Table 1). Most of their responses were expressed as questions; that is, students identified questions they were interested in learning about. We thematically clustered these questions, which are displayed in the “Questions Raised by Students” column in Table 1. The theme with the highest student interest (20.5%, *N* = 224) was the origin of COVID-19, referring to where and how COVID-19 emerged. The theme with the second highest student interest was vaccines (19.2%, *N* = 224), which included responses related to vaccine science, distribution, and safety. The third highest was transmission (16.5%, *N* = 224), or how COVID-19 spreads. When exploring if/how students’ interests might have change as the pandemic unfolded, only infection rate (i.e., how and why COVID-19 spreads so quickly) showed a significant difference in interest across the three time points (*p* = .011, Fisher’s Exact Test). Specifically, interest in COVID-19 infection rates decreased over time; 25.7% (*n* = 74) of students expressed interest in spring 2020, 14.1% (*n* = 92) in fall 2020, and 6.9% (*n* = 58) in spring 2021.

**Table 1:** Thematic Analysis of Student Interest Related to COVID-19

Theme	Questions Raised by Students	Exemplar Response	Percentage of Total Respondents (*%*, *N* = 224)
Origin of COVID-19	Where and how did COVID-19 emerge?	“Where it had originated from and how it came to be.”	20.5
Vaccines	How do vaccines work? How will vaccines be distributed? Are vaccines safe?	“I am interested in learning about whether there are any current medications/vaccines that can stop the disease before it gets too deep in the body, as well as if they can lessen the symptoms of COVID-19.”	19.2
Transmission	How does COVID-19 spread?	“What it is, how it spreads, that it’s not like the flu, asymptomatic cases.”	16.5
Infection rate	How and why does COVID-19 spread so quickly?	“I think it’s interesting how fast coronavirus has spread.”	16.1
Body’s response	How does COVID-19 affect the body and specific organs?	“How our body deals with it and the effects it has on our body.”	12.1
Biology of COVID-19	How do viruses like COVID-19 work? How do viruses replicate?	“How it works (what proteins/cells does it attach to/invade? Is it an RNA virus or a DNA virus?)”	11.6
Prevention strategies	How do we prevent spreading? How do we stay safe?	“High school students need to know how to prevent the virus from spreading to them. We should also know how to keep ourselves from spreading it to others, especially because people are age are normally asymptomatic.”	10.3
Symptoms	What are the symptoms? How long do the symptoms last?	“What interests me the most is how there can be so many symptoms.”	8.0
Individual response	Why does COVID-19 affect people differently?	“How it’s more dangerous to some more than others.”	7.1

### Information Sources

Information sources were clustered into seven categories as shown in Table 2 and commonly reported student examples of each theme are provided in the second column. Approximately 20% (*N* = 224) of the students surveyed used health agencies for information about COVID-19, which included sources such as the Centers for Disease Control (CDC), National Institute of Health (NIH), World Health Organization (WHO), and hospital websites. Other popular information sources included healthcare professionals (17%, *N* = 224), friends or family (14%, *N* = 224), TV news (13%, *N* = 224), social media (7%, *N* = 224), teachers (7%, *N* = 224), and online news articles (6%, *N* = 224). The proportion of students who reported using healthcare professionals for COVID-19 information significantly decreased throughout the course of the pandemic from 25.7% (*n* = 74) in spring 2020 to 14.1% (*n* = 92) in fall 2020 to 6.9% (*n* = 58) in spring 2021: *X*^2^ (2, *N* = 224) = 8.2, *p* = .017). The other themes did not significantly differ across the three time points.

**Table 2:** Thematic Analysis of Student Sources for COVID-19 Information

Theme	Example of Theme	Exemplar Response	Percentage of Total Respondents (*%*, *N* = 224)
Health Agencies	CDC, NIH, WHO, Mayo Clinic, hospitals, health institutions	“The sources that are the most important would include any government agencies and medical facilities.”	19.6
Healthcare Professionals	Doctors, nurses, other professionals in health fields	“Doctors and Health departments who know what they’re doing.”	17.0
Friends/Family	People in close contact with student	“Someone who actually has had first or second-hand experience with it. (Ex: my cousin was in the hospital for months dealing with the virus.) I would get information from her or her mother.”	14.3
TV News	CNN, FOX, CBS, NBC, ABC	“I would say cable news channels like Fox or CNN.”	13.4
Social Media	Twitter, Instagram, TikTok, YouTube	“I like to use social media like Instagram and Twitter for most of my information.”	6.7
Teachers	Classroom teachers	“My science teacher because she made an effort to inform us on the virus when we had school.”	6.7
Online News Articles	CNN, FOX, CBS, NBC, ABC, New York Times, Washington Post	“I scroll through my phone on Google and see posts from Washington Post or some other major news network.”	5.8

### Concerns

As shown in Table 3, the participants’ top concern about COVID-19 was contracting the virus or someone they know contracting the virus (20%, *N* = 224) followed by development and availability of vaccines (15%, *N* = 224). Students were concerned about multiple aspects related to vaccine production and distribution. One student responded that her greatest concern was: “When will we be able to get a vaccine and if the vaccine will also be made political and many people refuse to get it.” Students also reported concern about the COVID-19 death rate (13%, *N* = 224) and the effect of the virus on school (11%, *N* = 224). Some students specifically expressed concern about long-term school closures, lack of focus with online school, and college readiness. In addition, 8.5% (*N* = 224) of students reported concern related to prevention strategies, including social distancing, wearing masks, quarantining, and flattening the curve. Only concern about the economy significantly differed over time; concern about the economy was highest in spring 2020 (14.9%, *n* = 74), lowest in fall 2020 (3.3%, *n* = 92) and then slightly higher in spring 2021 (8.6%, *n* = 58) (*p* = .011, Fisher’s Exact Test).

**Table 3:** Thematic Analysis of Student Concerns about COVID-19

Theme	Description	Exemplar Response	Percentage of Total Respondents (*%*, *N* = 224)
Myself/Friends/Family Contracting COVID-19	Concerns about COVID-19 infection for themselves or others in close social circle	“My top concerns are getting infected and someone I know getting infected.”	20.5
Vaccines	Development, distribution, safety, and effectiveness of COVID-19 vaccines.	“How effective the vaccine will be and if people will be able to get the vaccine.”	14.7
Death Rate	Increasing death toll	“The amount of deaths by the end of this.”	13.0
School	Effect on student learning, online instruction, attention in school, motivation in school, grades, college preparation	“My education will be effected poorly due to changes in school, freshman year being incomplete and then the pains of online/hybrid/quarantined school, it’s effect on my college-readiness.”	10.7
Economy	Concerns about a recession, small businesses, the future market	“Economic impacts on small businesses.”	8.5
Prevention Strategies	Social distancing, wearing masks, quarantining, staying home, flattening the curve	“So many people are not social distancing and helping to flatten the curve that I worry for these people because they will be the ones most affected by this.”	8.5
Infection Rate	How quickly COVID-19 spreads, “waves” of infection	“How fast it is spreading.”	5.4

### Impact

The degree of the virus’s impact on daily life varied tremendously among individual student respondents. Several students experienced losses of lives (family members or friends), some expressed strong emotional reactions (feelings of isolation or mental health issues), while others reported little to no impact (17%, *N* = 224, See Table 4). The largest proportion of students responded that staying home had impacted their daily lives (22.3%, *N* = 224). Other students reported challenges with online school (11.2%, *N* = 224), effects on their jobs or their parents’ jobs (8.0%, *N* = 224), wearing masks (4.9%, *N* = 224), and people they know contracting COVID-19 (4.9%, *N* = 224). None of the themes significantly changed over the three survey time points.

**Table 4:** Thematic Analysis of Student Impact Related to COVID-19

Theme	Description	Exemplar Response	Percentage of Total Respondents (*%*, *N* = 224)
Staying Home	Limiting contact with others by remaining at home except for essential tasks	“We have been doing our part social distancing and staying inside. My family and myself included get very frustrated when we see people not following guidelines or not protecting themselves.”	22.3
Minimal Impact	Little to no impact other than following mandates such as wearing masks or social distancing	“Not affecting us much as none of my family has gotten it.”	17.0
Online School	Remote learning due to school closures	“I am doing schoolwork online but I don’t feel as if I’m learning anything. I feel trapped and as if the virus is going to cause a heavy increase of depression in my generation. There’s no point in online school since everyone is cheating or not even doing the work.”	11.2
Jobs	Changes in hours or pay, unemployment, remote work for student or their guardians	“My mom has to work two jobs now because her job doesn’t have full hours anymore because of COVID so she gets little sleep.”	8.0

## Discussion

The COVID-19 pandemic resulted in unprecedented times for students with drastic changes in school, routines, and everyday interactions. We identified student interest about various dimensions of COVID-19 at the beginning of the outbreak and throughout the duration of the pandemic to aid future teaching and learning about the virus as well as other potential outbreaks. Interest is of particular importance in the context of COVID-19 as it plays a central role in student motivation and learning. Specifically, engaging students’ situational interest, or interest sparked by environmental factors, is critical for the support of learning (Hidi & Renninger, 2006). Given the ubiquity of the pandemic (and presumably other similar disasters of this scale) and its necessary connections to science, it creates opportunities to trigger situational interest among students through classroom instruction even among students who may not have developed or demonstrated longer term personal interest in science and science learning. It is critical that all students understand the science behind COVID-19 and viral disease transmission to improve public health safety and help students make informed personal decisions related to the pandemic.

We sought to uncover the areas of interest to aid educators in curriculum design around the pandemic and glean additional information on information sources, concerns, and impact of the virus to contextualize student interest. We further discuss the results from our four research questions.

### Research Question 1

Research question 1 addresses the interests students have about COVID-19. The most reported themes in order from greatest percentage to least were the origin of COVID-19, vaccines, transmission, infection rate, body’s response, biology of COVID-19, prevention, symptoms, and individual response. The origin of COVID-19 remained of high interest to students across the three time points. Unsurprisingly, interest in vaccines reached its peak and was the highest percentage across all other themes in fall 2020 when the first COVID-19 vaccines became authorized for emergency use (Pfizer, 2020).

Compared to issues such as the virus’ origin and availability of vaccines, topics that tend to fit in traditional life science classes such as viral transmission, the biology of viruses, and human physiological responses were mentioned less frequently by the student respondents. This has important implications for educators and who want to build on students’ natural curiosities and interest in an emerging pandemic. Science teachers may see immediate connections between a viral pandemic and traditional curricular themes, such as the biology of viruses, and therefore be tempted to use these connections as a starting place for instruction. However, despite ostensible connections to the real-world issue, this approach may not effectively leverage student situational interest for prompting motivation to learn or the triggering of more enduring, individual interest (Hidi & Renninger, 2006). To meet student interest more generally, science educators should consider initiating instruction related to the pandemic with topics such as origin of the virus or vaccines, which were the two most popular themes students expressed. It is interesting to note that these two topics were at the center of debate the media (across many platforms) at the time of survey distribution. Therefore, the larger message (beyond teaching about the current pandemic) is a recommendation for educators to carefully consider the questions and controversies unfolding in popular culture as an entry point for issues-based teaching and learning particularly for emergent issues like a pandemic. This approach stands in contrast to what teachers often do when they try to incorporate SSI in their classes—prioritize connections to content ideas that stem from standards or traditional curricular approaches (Hancock et al., 2019). We are not suggesting that these connections not be attempted, but rather that these connections should follow efforts to build student engagement by tapping into aspects of the issue that students are more inherently interested in.

Across the world, conspiracy theories about where and how COVID-19 first emerged (i.e. human-made, bio-terrorism) and misinformation about vaccine safety (i.e. birth defects, infertility) have been spreading rampantly on social media (Roozenbeek et al., 2020). In fact, WHO coined the term “infodemic” to describe this problem. An infodemic refers to the overwhelmingly large pool of information during a disease outbreak that may or may not be factual and limits the ability to find reliable sources (WHO, 2021). Youth are especially susceptible to infodemics on social media platforms, where news is filtered by likes and dislikes rather than by factual content (Wiederhold, 2020). Our findings indicate that a significant proportion of students acquire information from people they know, TV news, and social media, which are often individually and politically biased. Addressing students’ interest about the origin of COVID-19 and vaccines in classrooms can help students develop scientific knowledge about viruses (Hidi & Renninger, 2006). Furthermore, discussions about these topics create opportunities to help students navigate the difficult task of discerning trustworthy information from the media they commonly consume, a skill that applies to the scientific practice of evaluating evidence.

COVID-19 presents a unique case where the science about the virus unfolded in real-time. Although infection rates of COVID-19 in the United States reached higher points than ever before in fall 2020, student interest in infection rates significantly decreased during this time (*p* = 0.01). A possible explanation could be that students became more knowledgeable about the spread of COVID-19 over the course of the pandemic. An alternative explanation could be “COVID fatigue,” a colloquial term used to describe the emotional drain as a result of constant stress and reminders of the pandemic. If these explanations were accurate, we would expect interest in other COVID-19 themes to decrease concurrently. Interest in transmission did decrease from 20.3% in spring 2020 to 15.5% in fall 2021, although this change was not significant. However, this trend was not the case across other themes. For instance, interest in prevention strategies slightly increased over the three time points and interest in the biology of COVID-19 peaked in spring 2021. While these trends were not significant, they highlight some students’ growing interest in aspects of the virus over time despite more knowledge or COVID fatigue.

### Research Question 2

Media consumption has become an integral component of personal experience among youth in the twenty-first century (Twenge et al., 2019), even more so during the COVID-19 pandemic when quarantining required individuals to access information and engage with society through digital means. We identified the most used sources of information about the pandemic among high school students as health agencies and healthcare professionals. Similarly, Smith et al. (2017) found that health organizations were the primary source of information among teachers. Only 6.7% of students in our sample reported using teachers as their source of COVID-19 information. Likewise, Campbell et al. (2021) found that high school students in semi-rural Georgia turned to peers and social media the most and schools the least for information about COVID-19. Integrating media platforms that students are interested in and frequently use in science classroom instruction is an avenue to encourage and sustain attention as students cultivate knowledge about the pandemic (Hidi & Renninger, 2006). Further, familiarity with popular information sources may provide students with increased sense of autonomy and competence (Hidi & Renninger, 2006), which are necessary as individuals contend with misinformation from the media, friends, or family. Students may also be more likely to remain invested in their learning even when they face challenges within a domain (Alexander, 2004).

In our study, one student explained, “I prefer to get information from my family though because it is easier to understand.” Given that websites like the CDC, NIH, and WHO are heavily trafficked by students and teachers, we echo Smith and colleagues’ suggestion that these organizations could better accommodate teachers and additionally recommend that information be organized in a comprehensible way for students for educational purposes. Science teachers are often reticent to introduce issues like pandemics into their classrooms because they can be controversial or uncertain (Hancock et al., 2019). Additionally, resources about viral outbreaks as seen with the Ebola crisis are limited, requiring teachers to do their own research and develop curricular material which takes time (Smith et al., 2017).

Due to school closures and increased time spent at home, we did expect students to consume more cable news than they would have otherwise. However, we did not observe any trends about the cable networks they reported watching. Some students were partisan in their viewership. For example, one student reported watching both CNN and Fox News, which tend to have opposite political leanings. Other students reported watching only one cable network like Fox News. During the study, the United States was in the midst of the 2020 presidential election. Politicians took to social media platforms like Twitter to disseminate COVID-19 information to the public, but this information was often biased or misleading. For instance, Donald Trump’s anti-vaccine tweets were found to heighten vaccine hesitancy among his voters (Hornsey et al., 2020). We found that high school students pay attention to political messaging. One student described following the governor of his state to acquire COVID-19 information. Several students reported using the president as their primary source of information, who was Donald Trump in spring and fall 2020 and Joe Biden in spring 2021. The COVID-19 information politicians publish on social media does permeate into high school students’ lives, which will influence the prior knowledge they bring into the classroom about the pandemic.

Across the three time points, students relied on healthcare professionals significantly less over time (*p* = .017). In fall 2020, less than a year since the first COVID-19 case emerged in Wuhan, China, there was still much uncertainty around the pandemic. Students relied the most on healthcare professionals for their source of COVID-19 information because of their firsthand experience with the virus. One student wrote she used the personal accounts of doctors and nurses “since they are the ones dealing with it and treating it.” However, in spring 2021 and fall 2021, the highest reported information source was health agencies. Over the course of the three survey time points, students reported using “government-funded, unbiased sources including the NIH and CDC to ensure that the information is current and reliable” more often than health professionals. This switch may have occurred as more health statistics were publicly available on health agencies’ websites for students to access. Furthermore, the high percentage of students using these health agencies demonstrates a sense of trust and perceived reliability of these sources of information.

### Research Question 3

The greatest concern to students was someone they know contracting COVID-19 or contracting the virus themselves. Students also expressed uncertainty about vaccines, fear of the death rate, and worry about their academic success with school closures. The main challenges students reported included increased time spent at home, loss of jobs, and the transition to online school, which have been linked to an increase in incidence of mental health issues (i.e. stress, anxiety, substance abuse) among youth during the pandemic (Thakur, 2020). Discussing topics related to the viral outbreak has been shown to reduce students’ worry about the pandemic (Ho et al., 2020). Thus, educators can relate these topics of student concerns with instruction about the virus. For example, student concern around vaccines, ranging from uncertainty about distribution of vaccines to their safety, increased at each time point. By spring 2021, 17.2% of students reported concern about vaccines. Teachers could integrate lessons about vaccines into science content related to the body’s immune response including topics such as active and passive immunity.

As stay-at-home orders began to lift and businesses started to reopen, students concern about the economy significantly decreased in fall 2020 (*p* = .011). However, as COVID-19 cases surged after winter holidays, students concern about the economy increased. Some students lost jobs; others described more family and childcare responsibilities at home while their parents worked. On the surface, concern about the economy may seem as though it does not relate to science instruction about the pandemic. However, the economic impacts that students experience at home do connect with scientific evidence demonstrating COVID-19 health disparities among marginalized and low-income groups (CDC, 2020). Furthermore, the third highest reported concern was related to the COVID-19 death rate. Teachers could help students think critically about factors that influence COVID-19 health statistics, including prevention strategies, access to health resources, accessibility of COVID-19 testing, and socioeconomic status.

### Research Question 4

We asked students to describe the ways in which COVID-19 has impacted their lives. While not all students experienced hardship during the pandemic—17% expressed minimal impact which included responses such as washing hands more often or as one student put it, “mild inconveniences”—other students expressed challenges with staying at home, online school, and jobs. Many high school students were expected to provide childcare or tutor siblings while trying to navigate online schooling for the first time themselves. Several students expressed highly emotional responses to the question, describing feelings of stress, grief, or social isolation. By asking about students’ personal experiences with the pandemic, we gained more insight into the difficulties students faced and how that might influence the interests they have in learning about the pandemic. We reported the top themes related to COVID-19 impact to raise awareness around the topics that directly relate to students’ lived experiences. Educators can use our findings by incorporating these personal connections to instruction about COVID-19. For example, students may be more interested in learning about the science behind wearing masks if it pertains to reopening business and students keeping their jobs. Students’ experiences, values, beliefs, and prior knowledge are a resource that teachers can use to sustain attention around science concepts related to COVID-19 and help students feel as though their thoughts and opinions are valued in the classroom (Gonzalez et al., 2005).

Although there were no significant changes in frequency of the themes across the three time points, we did notice a change in the number of students who reported knowing someone who contracted COVID-19. In spring 2020, none of the students reported knowing someone who had contracted COVID-19, but by fall 2020 and spring 2021, eleven students did. This may explain why peak student concern related to contracting COVID-19 occurred during fall 2020 as case numbers increased the probability of exposure to the virus.

### Limitations

One limitation of the study was that some students provided limited responses that were either vague or too brief to interpret. We could have asked probing questions to obtain more detailed and specific answers. For instance, many students wrote “social media” as their source of COVID-19 related information. An additional question could have asked what social media platforms students access. Additionally, future studies could ask questions that elicit explanations as to why students are interested in certain topics related to the pandemic. This information would help reveal more insight into student interest that could be connected to science classroom instruction.

## Conclusion

COVID-19 is a global threat that impacts everyone, creating the opportunity to relate science learning experiences to complex, societal issues that connect with students’ lives. We described major areas of student interest about the pandemic to better support student learning during classroom instruction about COVID-19. The goal of our study was to provide key areas of student interest about COVID-19 to help educators address students’ questions and concerns about the virus and improve SSI-based curricular resources on viral pandemics. In addition, we examined the sources that high school students use to acquire COVID-19 related information and its implications for media literacy and the spread of misinformation online in crisis situations more broadly. Connecting student interest, information sources, concerns, and personal experiences during the pandemic to instruction about COVID-19 can increase academic performance, content knowledge, attention, and engagement in learning about the virus and inform future efforts associated with pandemic- and other issues-based teaching.

## Data Availability

The datasets generated and/or analyzed during the current study are not publicly available due to them containing information about minors but are available from the corresponding author on reasonable request.

## Abbreviation

SSI: Socioscientific Issues
